# Seek and Ye Shall Not Find (Yet): Searching Clinical Trial Registries for Trials Designed With Patients—A Call to Action

**DOI:** 10.2196/72015

**Published:** 2025-05-30

**Authors:** Karen Louise Woolley, JD Woolley, Mark James Woolley

**Affiliations:** 1 Faculty of Health, Medicine and Behavioural Sciences, University of Queensland, St Lucia, Brisbane, 4067, Australia, 61 7 3365 7489; 2 Clinical Trials Centre, University of the Sunshine Coast, Sippy Downs, Australia; 3 Caregiver author, St. Lucia, Australia; 4 Patient author, Sydney, Australia; 5 Patient author, Sunshine Coast, Australia

**Keywords:** patient and public involvement, clinical trial, clinical trial registry, diversity, equity, and inclusion, patient author, caregiver author, patient partner, GRIPP2, plain language summary

## Abstract

Clinical trial registries were designed to help patients search for potentially suitable clinical trials. When our family faced another serious cancer diagnosis, we searched multiple international clinical trial registries. Despite increasing evidence that trials designed *with* patients can be better for trial participants (eg, they can have more relevant outcome measures and fewer burdens), it is currently impossible to search registries for these specific types of trials. In this Patient Perspective article, we make the first “call to action” for clinical trial registries to include (1) a filter that allows for efficient searching for clinical trials designed with patients and (2) structured information, in plain language, on how patients were involved. We propose how these two innovations could help reduce barriers to clinical trial participation. We also highlight how new regulatory and ethical guidelines are encouraging patient involvement in trial design, and we identify the benefits to many of doing so. Given the pressing need to improve clinical trial participation, we respectfully call on the clinical trial community to respond to our call to action and consider our proposed action plan. Ideally, when patients want to search for clinical trials designed with patients for patients, we should be able to find them. A plain language summary for this publication is available in the supplementary material for this paper.

When a serious cancer diagnosis struck our family—again—we searched clinical trial registries for trials designed *with* patients. Given the increasing evidence for the value of patient involvement in trial design, if we were going to consider a trial, we wanted to know if and how patients had been involved. Today, this search is impossible. In the future, we hope it can be routine. In this Patient Perspectives article, we provide the first published “call to action” for clinical trial registries to include (1) a filter that allows for efficient searching for clinical trials designed with patients and (2) structured information, in plain language, on how patients were involved. We propose that addressing these two gaps could accelerate clinical trials by enhancing clinical trial participation. We have included a plain language summary of this article in Multimedia Appendix 1.

Within our family, we have managed clinical trials, participated in clinical trials, and faced cancer diagnoses where our care has been directly enhanced by clinical trials. In our current situation, we already know we will be relying on evidence generated from forthcoming clinical trials. From these professional and personal experiences, we fundamentally understand that patient participation in cancer clinical trials advances cancer treatment [1,2]. However, for decades, most (92%‐98%) patients with cancer have not participated in clinical trials [1,2]. New ways to boost clinical trial participation are needed.

Importantly, when it comes to proposing potential solutions, we recognize that both nonpatient and patient barriers to trial participation must be taken into account. Notably, the main barriers occur well before a clinical trial is even offered to a patient [1]. That is, patients are not the main cause of low participation rates. The upstream nonpatient barriers can be structural (eg, access to a trial), clinical (eg, eligibility criteria) or doctor related (eg, offering a clinical trial) [1]. Indeed, when clinical trials are offered to patients with cancer, many (55%) agree to participate [1]. If and when a clinical trial offer is finally made to a patient, the patient may decline participation because of concerns related to treatment, trust, and the burden of participating [1]. In this traditional model, patients have not had an active and participatory role in finding clinical trials and in considering whether to participate. This traditional model can and should change. Our proposed innovations to clinical trial registries could positively disrupt this traditional model and help reduce both nonpatient and patient barriers.

In terms of nonpatient barriers, patients would not have to wait for clinical trials to “trickle down” to them through structural, clinical, and doctor-related barriers. Patients could have enhanced agency to find potentially suitable clinical trials designed with patients. They could find these trials more quickly, easily, cost-effectively, and independently via their own search of a clinical trial registry. For patients, self-searching for these trials, using a filter that matters to them, would be a new form of self-care. After all, it is patients who bear the greatest burden in a clinical trial. After patients found potentially suitable trials designed with patients, they could then work in partnership with their doctor to consider—from the medical and the patient perspective—whether to participate. Both perspectives can affect participation success (eg, recruitment and retention). As clinical trial registries were explicitly developed to allow patients to search for trials and as approximately half of registry users are patients [3], our call to action would help registries meet their original goals. Further, as anyone with access to the internet could search clinical trial registries, our proposal may also help break down diversity, equity, and inclusion barriers to clinical trial participation.

In terms of patient participation barriers, concerns about a trial may be reduced if potential participants knew that patients had been involved in trial design. Increasing evidence indicates that the “lived experience” from patient advisors can translate into a better “trial experience” for patient participants. For example, trials designed with patient input may be more clinically relevant, faster, less costly, and reduce the trial burden for participants [4-9]. Within our family, we have participated in patient advisory boards and have seen first-hand how patient input can enhance trial design. A protocol can go from good to great with patient input. If patients could access information on how patients had (or had not) been involved in a trial, we believe that this could affect their trust and interest in that trial.

Our call to action for a search filter and information on patient involvement in trial design aligns well with broader changes driving more involvement of patients in clinical research. For the first time, the Declaration of Helsinki, an internationally accepted and highly influential guideline on research ethics, now calls for researchers to involve patients meaningfully in trial design [10]. The ICH GCP (International Council for Harmonisation - Good Clinical Practice Guideline), issued by international regulators and adhered to by industry and nonindustry research sponsors, have recently been updated, with the new version explicitly calling for sponsors to involve patients in trial design [11]. Under the new European Clinical Trials Regulation, sponsors must also describe if and how patients were involved in trial design [12]. Importantly for both trial design and trial reporting, the new 2025 SPIRIT (Standard Protocol Items: Recommendations for Interventional Trials) [13] and CONSORT (Consolidated Standards of Reporting Trials) [14] guidelines now include specific items for reporting patient involvement in clinical trial protocols and publications.

If our call to action is taken up, patient involvement information in structured, plain language included in the clinical trial registry could build on the precedent set by *The BMJ* in 2014 [4]. To promote transparency and to avoid a tokenistic tickbox approach, *The BMJ* requires authors to include a patient and public involvement statement, which describes how patients were involved in the reported research. If the researchers did not involve patients, they must disclose that in their statement. As the patient and public involvement statement is included in the publication, readers (including patients) can readily identify if and how patients were involved. With more patients authoring publications [15], involving patients in trial design would make it more straightforward for these patient experts to meet authorship criteria. Further, transparency about early patient involvement would also facilitate research into the “patient advisor” to “patient author” journey. Given *The BMJ*’s intent to re-energize the Patients Included charter for conferences [16], we also encourage discussion as to whether the charter could extend to patients included in trial design. The earlier that patients and other stakeholders know about patient involvement in research, the better.

Without regulatory requirements and enforcement, a proposed change in clinical trial registry practices is unlikely to succeed unless key stakeholders see value in doing so. Our investigations have shown that the widely used registry ClinicalTrials.gov does not allow patients to search for clinical trials designed *with* patients; nor do other major registries managed by not-for-profit (0/18, 0%; Table 1) or for-profit (0/10, 0%; Table 2) organizations.

**Table 1.** Primary clinical trial registries in the World Health Organization registry network lack a search function for finding clinical trials designed *with* patients.^a^

World Health Organization: primary registries^b^	
Registry	Filter for patient involvement in trial design
1. Australian New Zealand Clinical Trials Registry	N
2. Brazilian Clinical Trials Registry	N
3. Chinese Clinical Trial Registry	N
4. Clinical Research Information Service (Republic of Korea)	N
5. Clinical Trials Information System (European Union)	N
6. Clinical Trials Registry - India	N
7. Cuban Public Registry of Clinical Trials	N
8. EU Clinical Trials Register	N
9. German Clinical Trials Register	N
10. Iranian Registry of Clinical Trials	N
11. ISRCTN (United Kingdom)	N
12. International Traditional Medicine Clinical Trial Registry	N
13. Japan Registry of Clinical Trials	N
14. Lebanese Clinical Trials Registry	N
15. Thai Clinical Trials Registry	N
16. Pan African Clinical Trial Registry	N
17. Peruvian Clinical Trial Registry	Site unavailable
18. Sri Lanka Clinical Trials Registry	N

a Registries were searched April 27 and 30, 2025.

b The World Health Organization lists 18 primary registries that meet its specific criteria; these registries also meet the requirements from the International Committee of Medical Journal editors [17].

**Table 2.** Clinical trial registries managed by major international pharmaceutical companies lack a search function for finding clinical trials designed *with* patients.^a^

Global pharmaceutical companies: clinical trial registries^b^		
Company	Company clinical trial registry	Filter for patient involvement in trial design
1. Merck & Co	Y	N
2. Johnson & Johnson	Y	N
3. Roche	Y	N
4. AstraZeneca	Y	N
5. Abbvie	Y	N
6. Bristol Myers Squibb	Y	N
7. Eli Lilly	Y	N
8. Pfizer	Y	N
9. Novartis	Y	N
10. Sanofi	Y	N

a Registries were searched April 27 and 30, 2025.

b Clinical trial registries managed by the top 10 global pharmaceutical companies (based on research and development expenditure in 2023) [18].

We recognize that resources would be needed to add a patient involvement search field to a registry and, ideally, to automate (eg, via human-in-the-loop artificial intelligence) the upload of patient involvement information from a protocol into a clinical trial registry. However, we anticipate that the benefits of these changes could outweigh the anticipated costs. For example, these changes might be paid for from the major financial benefits gained from increasing recruitment and retention, accelerating trial start-up and completion, and reducing protocol amendments and associated operational costs [5-8]. Additional benefits, across multiple stakeholders, could include the following:

- Acting as a catalyst for advancing truly patient-focused and patient-vetted research
- Providing the clinical trial community (including patients, researchers, sponsors, and ethics committees) with a free, fast, and transparent way to see how patients have been involved in trial design
- Enhancing the power and agency of patients to find and assess potentially suitable clinical trials, particularly for patients underserved by the current clinical trial enterprise
- Encouraging sponsors to use this tangible, transparent, and timely way to demonstrate how they have engaged patients as clinical trial advisors and how they have strived to enhance the clinical trial experience for participants
- Providing sponsors with a new and justifiable way to gain credit for their commitment to involve patients as research partners and to enhance their reputation among patients, the media, investors, and other communities
- Demonstrating to researchers and sponsors how they can leverage patient involvement content multiple times beyond registries (eg, patient involvement statements in protocols, grant applications, ethics submissions, publications, corporate annual reports, regulatory submissions, and reimbursement applications)
- Providing journal editors, reviewers, and readers with source information on patient involvement that can be validated and verified against protocols and publications
- Facilitating new ways to conduct research, undertake benchmarking, and identify best practices for patient involvement in trial design (eg, across trial type, phase, disease, country, or year)

As a family facing another serious cancer diagnosis, we are deeply grateful to all the patients, researchers, and sponsors who have and are enhancing cancer treatment through clinical trials. We respectfully call upon the clinical trial community, in its broadest sense, to consider the merits of enhancing clinical trial registries to enable patients to (1) search for clinical trials designed with patients and (2) find information on how patients were involved. From initial discussions within our family and, subsequently, with international thought leaders from patient advocacy, academia, publishing, and industry sectors, it appears our call to action has merit. We are now exploring how to move from a call to action to an action plan. While any action plan will require input from a broad stakeholder group, we propose that the following steps may help progress this initiative:

1. Share this open-access publication widely among the clinical trial community to build awareness of the call to action
2. Establish a small core team (eg, 3‐5 people representing different stakeholders, including patients) to help secure resources and develop a project plan, with short-, medium-, and long-term goals. Ideally, this core team would align itself with organizations already focused on patient partnerships and enhancing clinical trial design, trust, transparency, accessibility, and infrastructure (eg, the World Health Organization’s International Clinical Trials Registry Platform) [19]
3. Conduct stakeholder consultations with key representatives from clinical trial registry owners and clinical trial registry users, as well as experts in other core areas (eg, database architecture, compliance and security, artificial intelligence, user design, and plain language)
4. Conduct a “sprint” project (ie, time-boxed, iterative) to co-create proposed standards for a “designed with patients” filter and plain language–structured descriptors of patient involvement in trial design
5. Present results from the sprint to registry owners and identify registry owners (ideally, from not-for-profit and for-profit sectors) willing to pilot-test a prototype
6. Evaluate the results from the pilot tests against predefined criteria for success
7. Present and publish results from the pilot tests
8. If successful, advocate for broader implementation across international registries

We recognize that many steps will need to be taken to respond to our call to action, but this publication is a tangible first step. As our family was reflecting on how easy it is to use filters to search for and access information that can affect our lifestyles (such as cars, hotels, and flights), we pondered when it will be just as easy to search for and access information that can literally affect our lifespans. Because, when it comes to patient involvement in clinical trial design, we sincerely hope that one day our family can say to other desperate families, “Seek and ye *shall* find.”

## Supplementary material

10.2196/72015 Multimedia Appendix 1 Plain language summary (visual abstract).

10.2196/72015 Checklist 1 Guidance for Reporting Involvement of Patients and the Public 2 (GRIPP2) checklist.

## Abbreviations

CONSORT: Consolidated Standards of Reporting Trials
ICH GCP: International Council for Harmonisation - Good Clinical Practice Guideline
SPIRIT: Standard Protocol Items: Recommendations for Interventional Trials
