# Supplementary figures and images for: Seek and Ye Shall Not Find (Yet): Searching Clinical Trial Registries for Trials Designed With Patients—A Call to Action
Source: J Particip Med. 2025 May 30;17:e72015. doi: 10.2196/72015 (PMC12143847; doi:10.2196/72015)

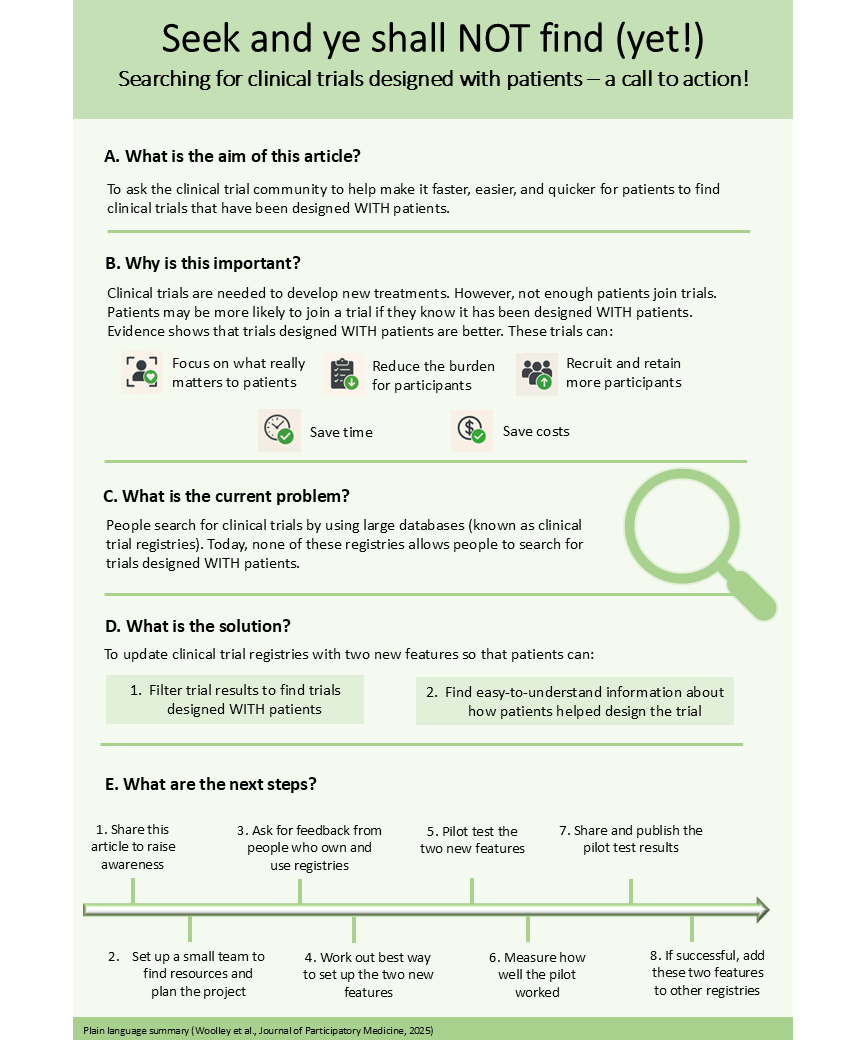

Supplement: Multimedia Appendix 1 [file jopm-v17-e72015-s001.png]
