# Supplementary material for: Seek and Ye Shall Not Find (Yet): Searching Clinical Trial Registries for Trials Designed With Patients—A Call to Action
Source: J Particip Med. 2025 May 30;17:e72015. doi: 10.2196/72015 (PMC12143847; doi:10.2196/72015)
Supplement: Checklist 1 [file jopm-v17-e72015-s002.pdf]

GRIPP2 short form

Woolley J et al Seek and ye shall NOT find (yet): Searching clinical trial registries for trials designed WITH patients

Section and topic	Item	Reported on page number
1: Aim	The aim of this paper, authored by two patients and one caregiver, was to make a 'call to action' to improve clinical trial registries.	3
2: Methods	This was a patient-inspired and a patient-informed paper. The patients and the caregiver were involved throughout the development of this paper. Input was gained from each author via emails, phone calls, and a face-to-face author interview meeting using a structured questionnaire.	6
3: Study results	The patients and the caregiver contributed to this paper by:<br>1. agreeing that we had come across an unmet need for patients and caregivers in the clinical trial environment<br>2. identifying and discussing the challenges experienced when trying to search for clinical trials designed with patients<br>3. considering individual perspectives (less and more experienced searchers) on how easy it should be to search clinical trial registries for patient-vetted clinical trials<br>4. suggesting how the proposed changes could benefit different stakeholders, and<br>5. making a commitment to prepare and review this 'call to action' paper even amidst personal health priorities	1-6
4: Discussion and conclusions	This paper would not have been considered without the personal experience of the patients and caregivers when they tried to search for trials designed with patients.<br><br>Given the challenges faced and the hope to raise awareness of the issue and make a positive change, a joint decision was made to aim to publish a paper to raise awareness of this issue.	3

Section and topic	Item	Reported on page number
	The ability to recognise and respond to this issue was enhanced by the collective experience of the caregiver and patients (eg, direct experience with involving patients in trial design, participating in clinical trials, relying on recent and ongoing clinical trials).<br><br>This is a ‘call to action’ paper. The proposed changes and actions will need to be informed by a broader stakeholder group.	
5: Reflections/critical perspective	The patient and caregiver authors were motivated to raise awareness of this issue after a recent cancer diagnosis. Although the catalyst for this work was personal, the authors believe the paper could help many others in the future.<br><br>By taking action now, the two innovations (ie, a filter for and information on patient involvement in trial design) may be ready when demand increases. If actions are not taken soon, then frustrations and defensive attitudes may take hold. As multiple stakeholders are already raising awareness of how patient involvement makes for a better trial experience, the demand for finding these trials is expected to increase. The authors recognise that changing clinical trial registries practices will take time, but that only intensified the urgency to publish the ‘call to action’ as soon as possible.<br><br>Focusing on this paper when trying to come to terms with a cancer diagnosis was challenging at times. However, working on the paper also provided a sense of hope and purpose that the ‘call to action’ could help others in the future.	6
